# A Simulation Study to Calculate a Structure Conceived by Eugène Viollet-le-Duc in 1850 with Finite Element Analysis

**DOI:** 10.3390/ma12162576

**Published:** 2019-08-13

**Authors:** Adela Rueda Márquez de la Plata, Pablo Alejandro Cruz Franco

**Affiliations:** 1 Department of Graphic Expression in Architecture, University of Extremadura, 10003 Cáceres, Spain; 2 Department of Construction, University of Extremadura, 10003 Cáceres, Spain

**Keywords:** simulation of construction, finite element method, linear and non-linear calculation, masonry dome, crosspiece, connecting rod, ductile cast iron, natural rock, Eugène Viollet-le-Duc, 1850

## Abstract

This study aims to investigate the application of finite element calculations to mixed structures of complex materials. As an example, we chose a vault designed by Eugène Viollet-le-Duc in 1850, at which time it was not possible to verify the complexities of the different materials working together in a single structure using these calculation methods. To carry out the simulation, the internal qualities of each material and its current equivalent are taken into account. Thus, the composition of each element is crucial for its integration into the whole structure and its modeling and subsequent calculation. With this research, we show that a finite element analysis can also be applied to structures that are yet to be built. Furthermore, we verify the technological, construction and materials knowledge that has led us here and demonstrate that what was once a utopian vision can now be realized using the structures and materials we have access to today.

## 1. Introduction

**Figure 1:**
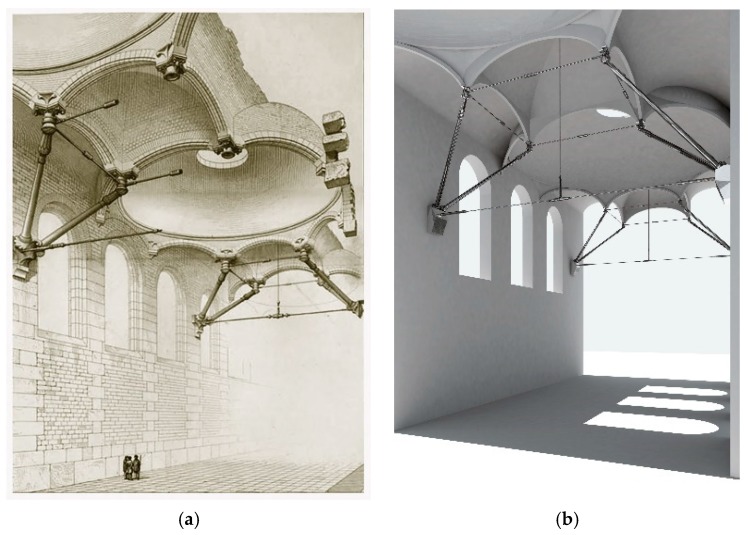
(**a**) Project proposed by Viollet-le-Duc for an ideal structure. (From *Entretiens sur l’architecture*, published in 1868). (**b**) Three-dimensional image recreated with AutoCAD + 3D Studio + Maya.

When Eugène Viollet-le-Duc published *Entretiens sur l’architecture* in 1863–1872, he proposed new structural ideas that combined classical models with domes and vaults, taking advantage of advances made thanks to new materials, such as the use of connecting rods and crosspieces. He also tried to create new spaces that were now needed by society, such as greenhouses, train stations, and large markets—spaces that were a direct cause or consequence of new technological advances and the Industrial Revolution (Figure 1a).

At no point in his theory does he speak about the theoretical variation in the spaces, but he does talk about a change in the proportions of the structures being used; in other words, a transformation from classical, traditional models to those that are altered by specific modifications and break away from structural language. The new structural model derives from the contradiction between the new structural language and the classical models on which it is based.

The chosen image shows a change in how architectural, structural, and constructive typologies are interpreted, and from this interpretation, a new concept of the structural model emerges. Therefore, there will be a conflict between the, generally modern, vision for technological understanding and new technology that transforms the presumptions and foundations that architects like Viollet-le-Duc based their work on. [1,2]

When it comes to typologies with large dimensions, emerging as the result of new architectural demands, the problem is the scale. The typology not only changes due to conditions of structural elements, but also, fundamentally, due to conditions of construction elements, which allow another level of scale to be reached and, therefore, may lead to a new typology being created.

The aim of the work is to demonstrate an incipient change in the conception of mixed structures of complex forms. It is considered that the starting point of this change begins with the structural utopia of Viollet-le-Duc in the mid-nineteenth century; in particular, the study is based on one of his most relevant and transcendent drawings. It is precisely this structure that has been completely digitized to build the model for the structural analysis presented below.

In this way, the present study assesses the possibility of using a calculation analysis with finite elements to compare the technological and working conditions between some utopian models of the past and new, more complex structures that would not be developed until much later.

To develop this finite element method (FEM) calculation, references and hypotheses already tested in high-impact research have been used. With regard to the behavior of masonry walls, the starting conditions and calculation method have been chosen based on those published by Italian researchers Marco Valente and Gabriele Milani with reference to the towers in the northeast of Italy [3] and in the study by the Italian research team Giuseppe Fortunato, Marco Franceso Funari, and Paolo Lonetti of the Baptistery of San Giovanni in Tumba [4].

However, the vault calculation has been modeled from the paper “Intuition, reason and calculation on the structural analysis of a ribbed vault” [5] and is based on what was written by Santiago Huerta [6] in “Arches, vaults and domes: Geometry and balance in the traditional calculation of factory structures.” In our opinion, these lay the foundation needed for structural FEM modeling of the utopian structure conceived by Viollet-le-Duc.

## 2. Methodology and Initial Considerations

The image was digitized and the general dimensions were taken from the image and an initial sketch made using AutoCAD. Then, a three-dimensional model was created in order to provide the same perspective as the original. Through this comparative process, errors in the proportions were adjusted and the project was geometrically defined.

We then reworked the AutoCAD plans using an inverse process to be able to create models of the floor, the raised levels, and other sections of the structure. Next, the modeling was finished in 3D Studio (Autodesk Inc in United States) to employ the materials being considered for structural calculations. Using the Maya program (Autodesk y Alias Systems Corporation in United States), the space was generically shaded to make it easier to discern the crosspieces and connecting rods that are part of the final image, which was otherwise very complicated (Figure 1b).

Once a useful model was developed, the process of structural dimensioning began with a small sketch of how the weight would be distributed across the model, paying particular attention to its strictly geometric function and determining which of the structural elements can function under compression or with traction.

The model derives from an illustration from which several conclusions can be extrapolated:

- The building has large dimensions. Representation of people inside the building’s gallery gives an idea of the project planning.
- The geometry that appears is repetitive, with multiple planes of symmetry. The central plane of the gallery is symmetrical, but so are the transverse vertical planes that pass through the point where the connecting rods meet the walls, and those that pass through the skylights.
- The roof is made using domes, with an indefinite surface that appears rotational.
- These domes are, in turn, connected via vaults, also with an indefinite surface, but they have straight guidelines.
- This roof is supported by arches that come out from the wall towards the inside of the gallery, meeting other parallel components at the same critical points of the structure, as said nodes meet the system of connecting rods and crosspieces.
- The set of arches forms an octagon/square sequence, where the octagon is taken up by a dome, and the square by a vault.
- It is considered that all the geometry designed by Viollet-le-Duc in the junction between rods and braces with the masonry wall has a structural behavior of the rotula type.

All of the geometric modeling explained in this study is a simplification, without losing sight of the fact that the final objective of the analysis is to verify the structural viability of the system from a global perspective, rather than the exact modeling of each piece.

## 3. Geometric Model

The following geometry was proposed based on the image, as has been previously explained:

- Gallery light: 19 m
- Height of gallery wall: 21 m
- Spherical dome:
  Development angle: 60°
  Skylight radius: 2 m
- Inner vault formed by the intersection of two cylinders:
          Development angle: 90°
- Lateral vault formed by one cylinder:
          Development angle: 90°

With these factors, the geometric solution is unique, except for the length of the connecting rods.

**Figure 2:**
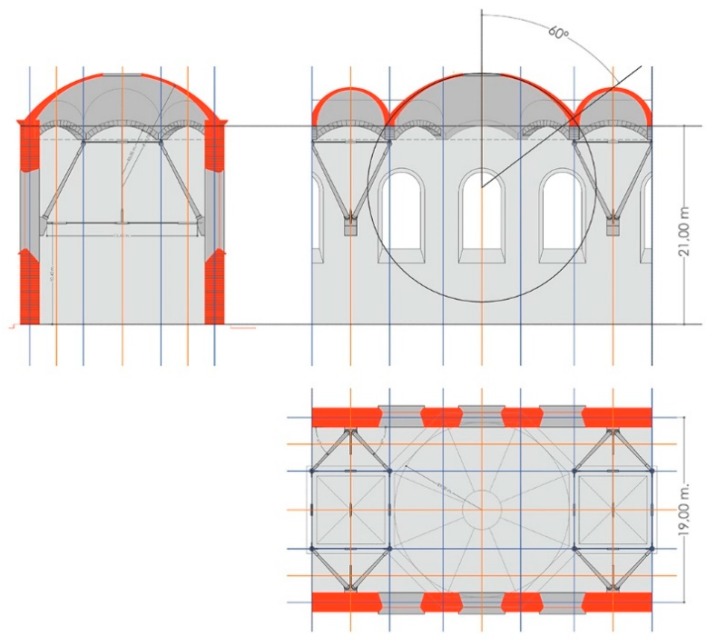
Geometric scheme used for the calculation from the original image.

The position of the connecting rod anchors on the wall varies, and will be a parameter to be optimized through modeling and structural simulation (Figure 2).

### 3.1. Geometric Interpretation

#### 3.1.1. System of Connecting Rods and Crosspieces

The system of connecting rods and crosspieces forms a mechanism that behaves like an isostatic structure under a particular type of load, such as its own weight [7].

The structure is an isostatic structure and, as such, the angle of its supports can vary with the deformations produced by the forces applied to it. The geometry of a static structure is adaptable and the supports on the ball joint avoid the transmission of torsion and bending stresses to the masonry wall, thus allowing the wall to be more slender and to operate exclusively with compression. The effort transmitted, in fact, by the combination of a rigid structure (walls and vaults) and a static structure (connecting rods and tie rods) allows each element to work in the most favorable way, achieving the global viability of the structure. This design avoids the auxiliary structures of flying buttresses and the buttresses of the masonry structure, and gives the feeling of weightlessness and lightness that Viollet-le-Duc conceived in its structure.

**Figure 3:**
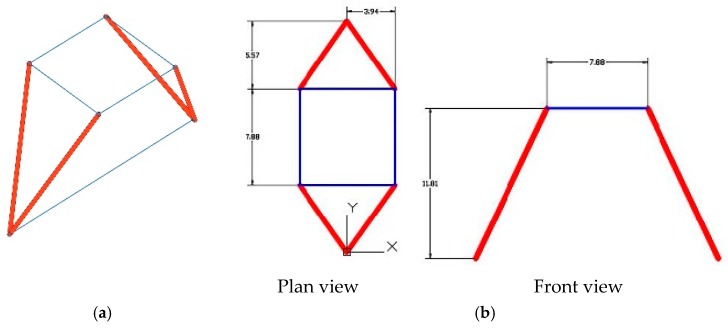
(**a**) Three-dimensional geometric diagram of the connecting rods and crosspieces; (**b**) geometric diagram of the system of connecting rods and crosspieces working together.

The final configuration chosen is that shown in Figure 3. The forces induced by the different structural elements are the force of the arch in the transversal direction to the gallery and the force of the dome.

It is important to clarify that, in this section, and as a previous geometric interpretation, only the thrust of the arch in the transversal direction of the gallery and the thrust of the cupola will be taken into account. This selection of forces is considered the most appropriate to begin a pre-dimensioning of the structure in order to enter the data into the SIMULSOFT program and begin FEM analysis.

#### 3.1.2. Force of the Longitudinal Arch

**Figure 4:**
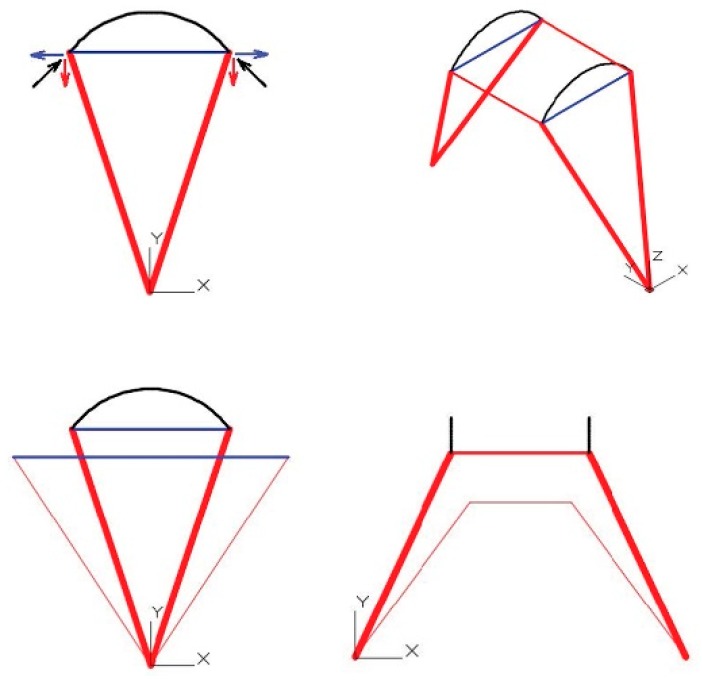
Diagram of functionality of the longitudinal arch thrust.

In Figure 4, drawn in blue is the crosspiece, which will be under traction due to the arch. The other elements will remain under compression. The elements under compression are those that bring loads towards the wall by way of the arch [8].

This effort will cause the overall structure to move down due to

- Lowering through widening of the transversal crosspiece;
- Lowering through shortening of the connecting rod and the longitudinal crosspiece.

#### 3.1.3. Force of the Dome

The dome will exert a vertical force corresponding to its weight, plus a horizontal force depending on the development angle of this same element.

**Figure 5:**
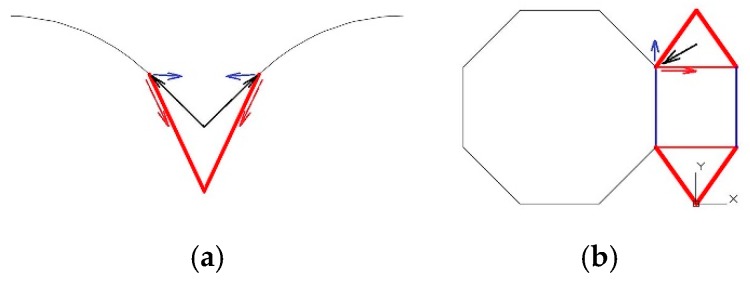
(**a**) Diagram in section view of the operation of the vault thrust; (**b**) diagram in plan view of the operation of the vault thrust.

This total force (vertical plus horizontal) seen in the vertical plane, which contains the connecting rod, will depend on Figure 5a. When seen from above, this will depend on the induction of compression in the red crosspiece and traction in the blue crosspiece (Figure 5b).

The tensioned braces are those that are responsible for counteracting the thrust of the dome “uprising it” against the reverse movement produced by the compression rod [9].

### 3.2. Other Things to Consider

The system of connecting rods and crosspieces is less rigid than the lateral load-bearing wall in the gallery. This hypothesis is taken as the starting point of the investigation. As this is a theoretical structure, we have had to create limits. One of them is this affirmation.

Its lower level of rigidity with regards to the wall means that the support of the dome with this structure moves down further than the wall does. This lack of symmetry will produce damaging forces in the dome. This could be thought of as a significant increase in the connecting rods and crosspieces, but this is not possible due to:

- Cost of materials. Elements, especially the connecting rod, will be extremely heavy and extraordinarily expensive. In addition, the installation may be completely unfeasible.
- Reinforcement of the dome. The more rigid the system, especially the connecting rod, the more the dome will undergo localized deformation in the most pronounced support area.

Although it is predictable that a suitable geometry of the connecting rod–crosspiece system would be beneficial, the overall rigidity of the building’s structure should always be taken into account. Therefore, different geometrical configurations must be studied, for which the only parameter is the height (the remainder are dimensions derived from other building factors) and the relative rigidity, for which the only parameters are the sections of the elements.

## 4. Construction Materials Used in the Model

Three construction materials were used in this model: natural rock, mortar, and ductile cast iron.

Natural rock is composed of fine-grain limestone and is used for the construction of walls and roofs. To simulate the behavior of the masonry walls, the characteristics of the mortar connections must be considered, which are the weakest found in the structure.

The aim of modeling the mortar is to provide lamination guidelines for the parts built from stone, especially the roofs. The chosen lamination model is Mohr–Coulomb. This material will mark the upper limit of acceptable tension.

Ductile cast iron is used to build the system of connecting rods and crosspieces. 

We can see in Table 1 the evaluation of construction models:

It is important to include certain clarifications on the characteristics of the materials for their subsequent FEM calculation.

Masonry is a material that has different properties depending on the direction in which the mortar joints are oriented. These constitute planes of weakness. The failure of the masonry structures is generally preceded by a massive development of cracking in these joints, so these limit the final resistant capacity. Some of the properties that have been taken into account and that characterize the masonry are the following. The mechanical strength of the masonry is extremely low while the mechanical union is low, and in design calculations it is usually taken as 0. The behavior of the masonry subject to complex tension states is markedly influenced by the orientation of the mortar joints and the applied loads. Most of the plastic deformations appear in the joints, and the characteristics of these are affected by the magnitude of the shear and the normal tension. Depending on the degree of compression to which it is subjected, failure can occur only in the joints or in a joint–mortar cracking combination. Not all fracture mechanisms in masonry are completely understandable and the use of plasticity criteria applied in geomaterials is usual (Mohr‒Coulomb).

## 5. Pre-Dimensioning of the Main Elements

### 5.1. Pre-Dimensioning of the Dome

The main tensions in the dome do not depend on its thickness, but on the density of the material.

**Figure 6:**
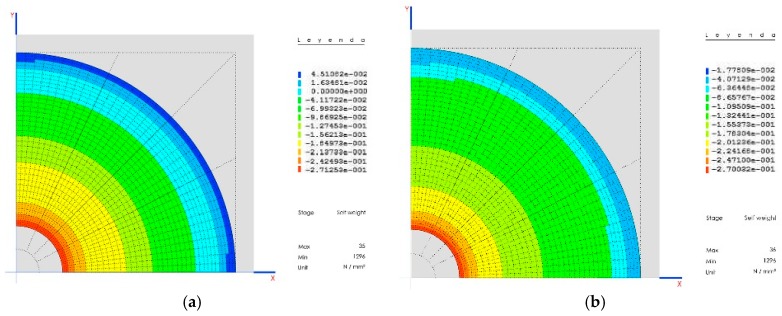
(**a**) Parallel stress; (**b**) the whole dome working in compression.

In this way, the tension in the meridians is Nϕ es: Nφ = d R/(1 − cosφ); and the tension in the parallels is Nθ es: Nθ + Nφ = R·d·cosφ; where d is the density of the material, R the radius of the sphere, and ϕ the angle measured from the upper pole of the sphere (upper pole = 0° halfway point = 90°) (Figure 6a). For the model data, these are: [10]

- R = 10.97; d = 2850 kg/m^3^; ϕ = 60°;
- Nϕ = 65529 kg/m^2^ = 0.625 MPa compression;
- Nθ = 49896 kg/m^2^ = 0.499 MPa traction.

To eliminate traction from the parallels, a change occurs in the sphere, in such a way that the normal load increases according to the growth of the dome. For the model, the dome has been divided into three thicknesses, 15, 30, and 50 cm (Figure 6b).

### 5.2. Pre-Dimensioning of the Arches

Assuming an initial thickness e = 15 cm for the dome, its total weight will be

(1) Q = d · Vol = d · (2· π · R · h · e).

Taking h as the central curve of the dome, h = 5.48; this way, the total weight of our dome is: Q = 161,474 kg.

Each arch supporting the dome will carry a proportional weight, 1/8, so the total weight of each arch is Qi = Q/8, which in our case is: Qi = 20,184 kg = 202 kN.

Assuming that the distribution of said weight is across the whole length l of the arch, with l = 7.87 m, the distribution will be: q = Qi/7.87 = 25.6 kN/m

The curve of the arches is a = 1.41, so that the horizontal force H is

(2) $$H\;=\;\frac{q\;\cdot \;l}{4\;\cdot \;f}\;=\;35.72\;\mathrm{kN}.$$

The vertical force at the extremes V will be equal to q/2 = 12.8 kN.

The section the arch must support, due to the weight of the dome with a total normal force, is N = 36.6 kN.

The area A that is strictly necessary to support force is A = N/fck = 36.6 kN/200 MPa = 183 mm^2^.

A section of 1 × 0.5 m will be taken, due to the increase in weight, and to be able to physically hold the meeting point of a dome with a thickness of 50 cm.

### 5.3. Pre-Dimensioning of the Walls

The weights that reach the wall from the dome are as follows:

For the weight of the dome, we assume the fourth part reaches Q/8 = 162.5 kN = V1

The lateral force will be H1 = 0. These moments have not been considered because during the construction phases (fully explained below), the crosspieces for eliminating horizontal thrusts are positioned before building the arches and, therefore, the domes. Thus, the domes cannot provide any destabilizing moment to the walls.

We take a resistant length of 12 m of the wall length (65% of the gallery light), in which the large 4 m windows are found, separated by 4 m. We have an 8 m resistant section.

We begin with an initial wall thickness of 2 m.

The momentum due to the vertical weight V1 on the wall is V1 = 325 × 1 = 325 kN·m.

For the weight of the wall, and the section above 2.7 m in height, Ps = 12 × 2 × 2 × 7 × d = 1846.8 kN.

For the section between the large windows, 9 m in height, Pi = 8 × 2 × 9 × d = 4104 KN.

The total weight of wall is 5950.8 kN.

This weight generates a stabilizing momentum of 5950.8 kN.

**Figure 7:**
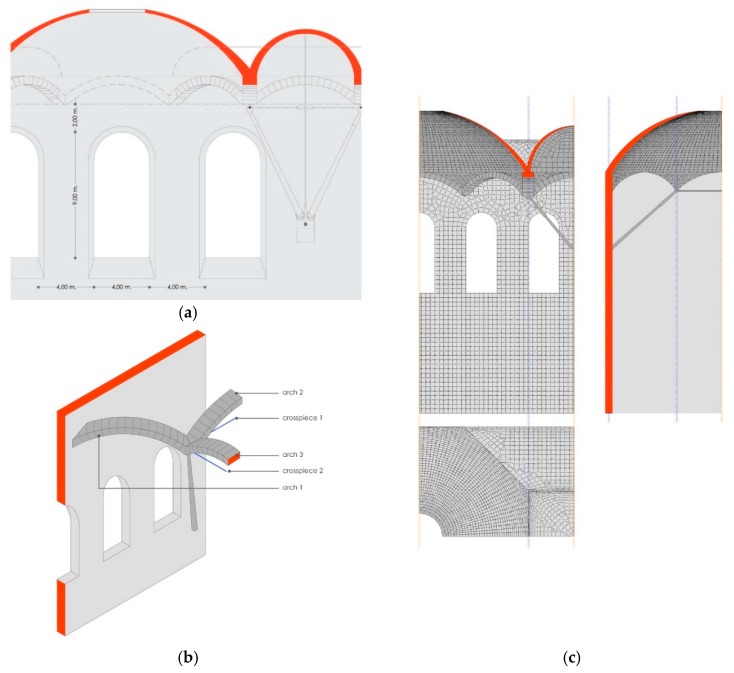
(**a**) Diagram of the proportions and measurements for the development of the three-dimensional model; (**b**) name of the structural elements for the model and for this study; (**c**) diagram of the connecting rod–arch support.

In total, the stabilizing momentum is 6275.8 kN·m against a destabilizing momentum of 325 kN·m (Figure 7a). In other words, the structure is totally safe against overturn.

## 6. Three-Dimensional Modeling for Elastic Calculation

Due to the symmetry in the structure, only part of it is considered, as the SIMULSOFT program [11,12] can account for symmetry in both the geometry and the weights [13].

Each element has been named independently as follows (Figure 7b):

- Arch 1: originating from the wall;
- Arch 2: parallel to the gallery axis;
- Arch 3: transversal to the gallery;
- Crosspiece 1: parallel to the gallery (longitudinal);
- Crosspiece 2: transversal to the gallery.

All stone elements are modeled using plate elements, except for the arches, which are modeled with beam-style elements [14].

The crosspieces and connecting rods are modeled using bar-style elements (joined at the ends).

### 6.1. First Model

The first model only consists of elements built from stone, without the system of connecting rods and crosspieces (Figure 7c), considering the following: Table 2

- Weights that reach the support between the connecting rod and the arches;
- Optimum position of the connecting rod;
- Vertical movement of the wall following loading;
- Dimensioning of the connecting rod.

#### 6.1.1. Forces on the Support and Position of the Connecting Rod

If the connecting rod is positioned in said direction, the work done by said structural element will be used as much as possible. The angles that make up the resultant are

- Horizontal = 47.57° with regard to the X axis; and
- Vertical = 78.19° with regard to the XY plane.

The horizontal angle cannot be geometrically reached due to the position of the wall with regard to arch 2 and its length, given that it is essential for the connecting rod to have the horizontal factors of X = 3.935 m and Y = 5.569 m, which translates into a fixed horizontal angle of 54.7530°.

With regards to the third factor, this does allow optimization of the position of the meeting point between the connecting rod and the wall, with one limitation: namely, the ground.

The reactive elements in the following directions are given in Table 3.

For the optimum angle of 78°, there is no point on the wall, but there is on the floor of the gallery. So, we dismiss this solution.

The angle of 45° generates forces that must be absorbed by other structural elements (the crosspieces), but due to their quantity, some dimensions similar to the connecting rods will be required, which is why this solution is also dismissed.

Therefore, we chose the solution of the connecting rod set up with an angle of 60° to the horizontal plane.

We can see in Table 4 the used dimensions:

However, definitively, a point has been taken, which means that the connecting rod forms an angle of 59.9949° with the horizontal plane. 

We can see in Table 5 the different components:

#### 6.1.2. Displacement of the Wall and Dimensioning of the Connection Rods

We can see in Table 6 the displacement of the wall:

A connecting rod in the solid circular section, with sufficient rigidity to experience the same vertical shifts DZ, would have a section of

(3) $$S\;=\;\frac{\mathrm{FZ}\cdot L}{E\cdot 0.147\;\mathrm{mm}}\;=\;\frac{1205\;\mathrm{kN}\cdot 13635\;\mathrm{mm}}{175\;\mathrm{GPa}\cdot 0147\;\mathrm{mm}}{=\;638,685\;\mathrm{mm}}^{2}.$$

This entails a diameter of 902 mm. The weight of the connecting rod would be around 653 kN, i.e., 65 tons. The rigidity would be

(4) $$\frac{E\cdot S}{L}\;=\;\frac{175\;\mathrm{GPa}\;\cdot \;638685{\;\mathrm{mm}}^{2}}{13635\;\mathrm{mm}}=\;6719\;\mathrm{kN}/\mathrm{mm}.$$

By decreasing the diameter to 315 mm, we obtain a section of 77,931 mm^2^, with a weight of 79.7 kN, i.e., 8 tons. The rigidity would be E·S/L = 1000 kN/mm.

This would mean that the connecting rod support has a curve of approximately 0.147 mm·6719/1000 = 0.987 mm (without taking into account the effect of the weight of the connecting rod or other forces).

The critical bending weight is more than 4800 kN.

### 6.2. Second Model

The following model is identical to the previous one except that the connecting rods and crosspieces have been activated (Figure 7c). For the connecting rods, we have already selected their dimensions. The dimensions of the crosspieces still need to be found.

The system of connecting rods and crosspieces is a mechanism, but due to the double symmetry, it behaves like an isostatic structure. This means that the shortening/lengthening movements produced on any of the elements that make up the system do not generate tension for the others, although they do generate movement.

The movements of the support between the connecting rod and the arches have again been blocked. On this simple support, movements of 1 mm have occurred in the three spatial directions, X, Y, and Z. With this, it is possible to calculate the rigidity of the set and determine the dimensions of the connection rods. 

We can see in Table 7 the different reactions:

This matrix of rigidity, K, allows us to anticipate the movements produced by a determinate force. Any force introduced generates movements in the three directions.

In this way, a force Q on the support, of the components QX QY QZ will produce some K−1•Q movements: K−1 × (173 195 −1298) = (−0.3686 mm 0.0449 mm −1.3189 mm).
For example, upon releasing the support between the connecting rod and arches, movements will be produced, namely, (73 95 −1298), an equal and opposite force to the reaction on the support.
If we then exercise a force that is equal to what results on the support between the connecting rod and arches, this will return to its initial position. These forces may be introduced by the crosspieces.
If we consider significant crosspieces on the arches of 124 mm diameter, these have a rigidity of 537 kN/mm. If they are shortened by 1 mm, they exercise a force (in the direction of X and Y) of 537 kN, which translates into different movements, as shown in Table 8.

However, as there are only two crosspieces, we can consider:

- Preventing the point from moving in two dimensions XY, YZ, ZX; however, the third degree of freedom will remain free and will produce extra forces on the structure.
- Optimizing the new position, for example, minimizing the distance between the initial and final position of said support.

For the first point, some tensions must be implemented equal to

(5) $$573\;\frac{\mathrm{kN}}{\mathrm{mm}}=\;{\left[\begin{array}{cc} 0.35411 & 0.088639\\ -0.08839 & -0.28916 \end{array}\right]}^{-1}\cdot \left[\begin{array}{c} 0.3686\;\mathrm{mm}\\ -0.0449\;\mathrm{mm} \end{array}\right]=\left[\begin{array}{c} 625\;\mathrm{kN}\\ 270\;\mathrm{kN} \end{array}\right].$$

The positive tensions are tractions, so it is possible to implement them with crosspieces (the compressions make them in danger of bending).

Some crosspieces with dimensions of 7.87 in length may make the system be loaded with variations in the usual temperature, depending on the coefficient of thermal expansion of the lot and the difference with smelting.

This has a coefficient of 1 × 10^5^ °C^−1^, while the limestone rock is around 1.2. An increase in temperature of 25 °C (from summer to winter) would cause shortening of (1.2 −1) × 10^5^ × 25 × 7870 = 0.4 mm, which is a load of 214 kN.

This is why we will choose some crosspieces that are 10 times less rigid. This also has the advantage that, when they are tightened, controlling their distortion can be done with greater precision. A very rigid crosspiece demands small movements that are more difficult to control.

The diameter of the crosspiece will be 40 mm and its rigidity will be 56 kN/mm.

### 6.3. Third Model

With this model, we want to observe the tensions produced on the arches and on the points where compression is minimized, which should not necessarily be in the key, due to the differential centers and the movements at the ends.

They include the aforementioned dimensions for the connecting rod, namely 315 mm in diameter and 40 mm for the crosspieces, and we will compare the results with crosspieces of 120 mm.

In this way, the following tables show the worst axial forces on the arches.

**Figure 8:**
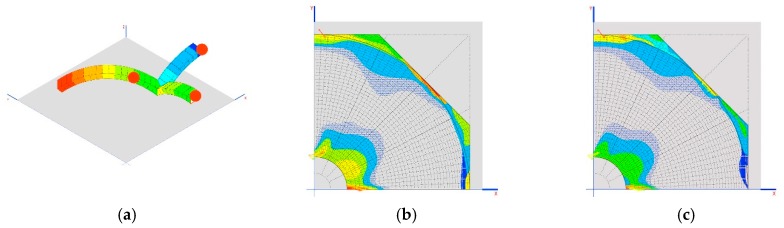
(**a**) Situation of the worst sections for the axial stress of the structural stone arches; (**b**) location of traction in the dome for the 120 mm crosspiece, up to 0.852 MPa; (**c**) location of traction in the dome for the 40 mm crosspiece, up to 0.856 MPa.

The cases of Prestressed_1 and Prestressed_2 weight are in the case of reductions of 1 mm on each crosspiece (Figure 8a).

Another interesting result is the tensions generated on the dome as demonstrated in the images shown in Figure 8b,c.

Analyzing Table 9 and Table 10, it can be proven that the three arches cannot be compressed at the same time. Only two can be compressed, namely, 2 and 3 (they are the only ones that undergo compression when the crosspieces are shortened). To compress the first arch, however, it is necessary to lengthen the crosspieces, which is only feasible if these are prestressed.

The results of the tensions of the dome indicate that it is not working in a compatible manner, but that, due to the movements produced in the elastic system, it starts to generate other forms of work and resistant mechanisms.

Finally, we observe movements produced on the support between the connecting rod and arches, as shown in Table 11.

There are at no time large movements. The structure is very rigid. This also allows us to generate areas of lamination that adapt to the geometry to allow the dome to work more efficiently.

### 6.4. Other Models

To continue with the investigation, other calculation models have been made by varying certain parameters.

**Figure 9:**
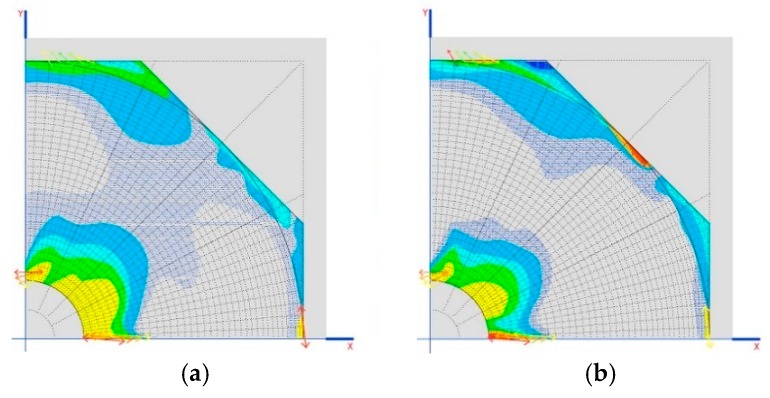

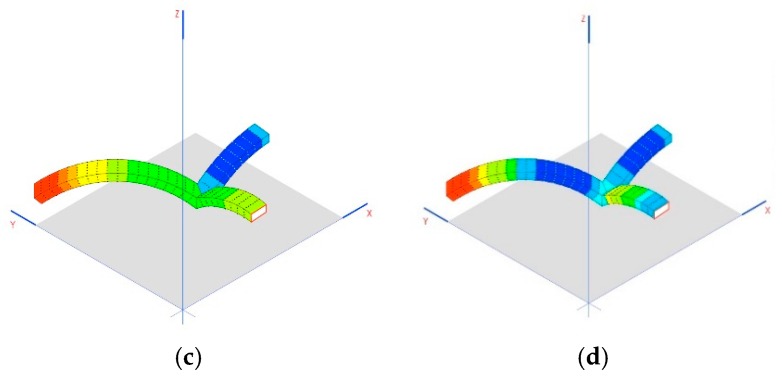
(**a**,**c**) Tractions in the dome with connecting rods at 45°; (**b**,**d**) traction in the dome with connecting rods at 60°.

As we can see in Table 12, we develop a comparison of the tensions produced in the dome and forces in the arches for connecting rods at 45° and 60°, with crosspieces of 120 mm (Figure 9a,b).

The forces on the arches are shown in Figure 9c,d. 

We can see in Table 13 the displacement in the support:

We have around half the forces and movements, so this is a good optimization.

Adding a crosspiece at the base of the connecting rod, as shown in the image of the original lithograph, does not significantly improve the model (less than 1% for forces and movements). This is due to the fact that the chosen anchorage point for the connecting rod on the wall is low, meaning that the wall has very little movement and it is not necessary to introduce said crosspiece to absorb the horizontal impact of the connecting rod.

## 7. Three-Dimensional Model for a Non-Linear Calculation

To execute a non-linear model, the following factors will be set up for the materials:

- The stone material plasticizes, according to a Mohr–Coulomb model, not with the parameters of the limestone, but for the connection material, which will be a type of mortar.
- The smelting material will not reach its elastic limit, and it will not plasticize or generate large deformations.

In such a way, the results of the model must remain of a plastic design with small distortions [15,16].

### 7.1. Fourth Model

This is similar to model 3, but some important variants have been introduced. The aim of this model is to observe whether the dome can remove tensions through plastic distortion, without incurring instability.

These tensions are up to 0.85 MPa, greater than 0.3 MPa, which supports the chosen mortar.

#### 7.1.1. Stone Material

This follows the same elastic and density properties. The lamination criteria that have been chosen are those of a mortar for connections, with a breaking tension of fck = 7.5 MPa under compression and ftk = 0.3 MPa for traction. With these values, the parameters of the Mohr–Coulomb model are:

- C = 0.75 MPa for cohesion; and
- Φ = 67° for the angle of internal friction.

#### 7.1.2. Plate Elements

For the plate elements that form the roofs, neither their flexion resistance or shear strength will be considered outside their planes, so as not to generate mechanisms that are different to those of a dome (or vault) [17].

Eliminating this resistance always leaves us on the side of security

### 7.2. Results

**Figure 10:**
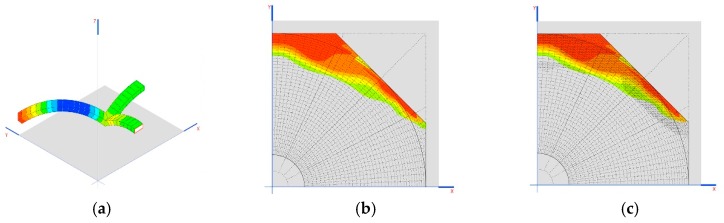
(**a**) Stresses in the arches; (**b**) traction stresses MPa; (**c**) plasticized areas (shaded area).

According to this model, the structure has sufficient lamination capability so as not to incur instability (the model is convergent) and to adapt to the movements produced by the loads (Figure 10b,c). This highlights the relaxation effect on tensions in the skylight area, whose traction tensions may not be considered void. However, this relaxation in the dome does not impede arch 1 from continuing to be subject to significant traction forces, and even arch 2 has traction. This form of work is incompatible with a piece of stonework (Figure 10a). The forces on the arches must be corrected through an appropriate construction process.

## 8. Simulation of Construction Phases in Linear Analysis

**Figure 11:**
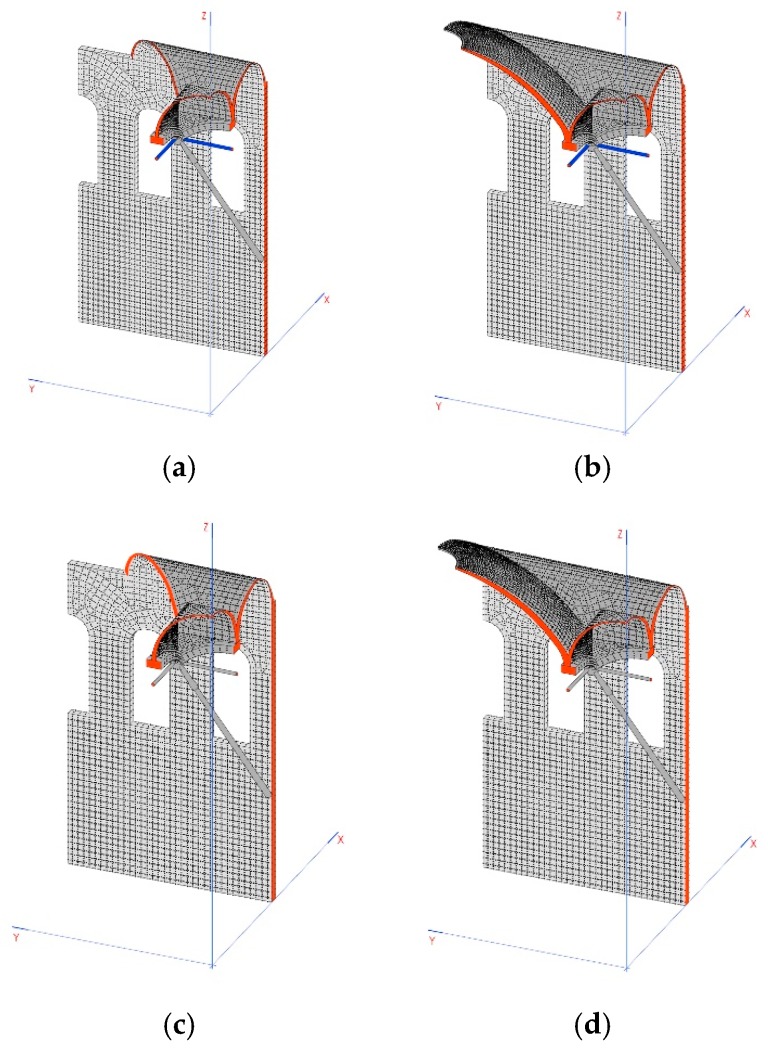
Diagrams of the different phases of construction: (**a**) gallery, (**b**) tension, (**c**) dome, and (**d**) relaxation.

The defined construction phases are as follows:

- Gallery: construction of walls, arches 2 and 3, system of connecting rods and crosspieces and the vaults that are between the domes (Figure 11a).
- Tension: implemented using the crosspieces to compress the arches (Figure 11b).
- Dome: construction of arch 1, the dome and the other closure elements (Figure 11c).
- Relaxation: releasing tension from the crosspieces to distribute the weight between the arches (Figure 11d).

### 8.1. Limiting Tension

The tension elements are adaptable function crosspieces, which allow maximum lengthening of up to 12% before breaking.

If the elastic limit is 175 MPa, the maximum load for the crosspieces of 40 mm diameter is N = 220 kN.

### 8.2. Tension Load

For crosspieces of 40 mm, we would obtain a maximum traction in arch 2 of 116.4 kN. If we want to compress it up to −100 kN, we need to introduce −216.4 kN of force. 

We can see in Table 14 the different forces:

However, it is not the structure that we have to compress, but the first phase of construction.

### 8.3. System Rigidity in Phase 1

Applying the elements of phase 1, the self weight and tension of 1 mm shortening of the support between the connecting rod and arches is shown in Table 15.

As with arch 1, which we want to compress in the final phase, positioning it at 45° is a good strategy for introducing the same tension load on arches 2 and 3, so that at the moment of releasing this tension, it is sent in the direction of arch 1.

In the case of the 120-mm crosspiece, we can tighten by 1 mm on crosspiece 1 and 0.2 mm on crosspiece 2, which generates 533 kN on each crosspiece. Finally, arch 2 will be 227.6 − 1 × 435.9 + 0.2 × 16.2 = −205.1 and arch 3 will be −145.4 + 1 × 9.6 − 0.2 × 535.2 = −242.8 kN.

In the case of the 40-mm crosspiece, we can tighten by 7 mm, which generates 392 kN (these elements have a rigidity of 56 kN/mm). This exceeds the maximum load of 220 kN that we have implemented. 

We can see in Table 16 the influence matrix loads:

We can tighten by 3 mm and 513 kN on crosspiece 1, and by 1 mm on crosspiece 2, keeping both arches compressed: arch 2 at −234 kN and arch 3 at −397.2 kN.

For a 70-mm crosspiece, the flexing weight is 125 kN. No crosspiece can exceed said weight. With a security factor of 2, we fix the maximum acceptable compression at 62.5 kN.

These tensions will relax in proportion to how the set distorts when introducing the real tension of the bars. It is an approximate calculation that will need to be confirmed when executing the complete model during the construction phases.

### 8.4. Results

It is shown in Table 17, Table 18 and Table 19 the different results of displacement and axial forces:

Crosspiece 2 exceeds the acceptable flexing weight fixed at 62.5 kN. We do not relax crosspiece 2 in the final phase of relaxation.

**Figure 12:**
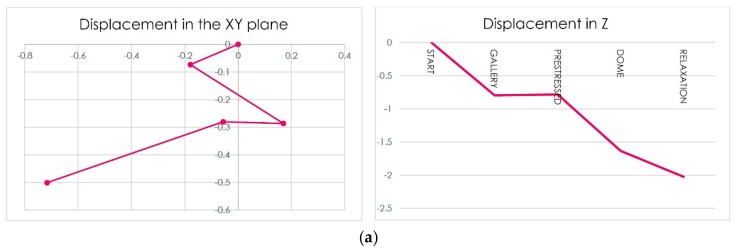

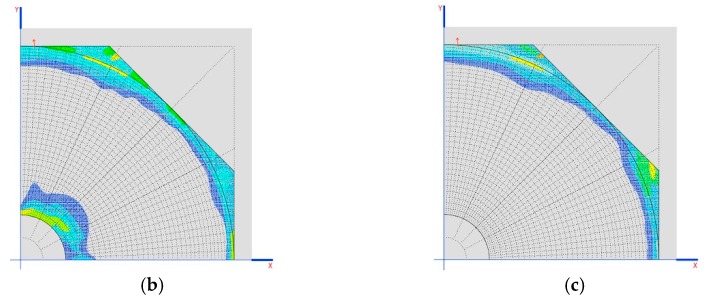
(**a**) Scheme of displacement of the point of anchorage of the connecting rod with the arches; (**b**) traction in the dome (MPa) in the penultimate phase of construction; (**c**) traction in the dome (MPa) in the last phase of construction.

Traction on the dome (MPa) in the two final phases is shown in Figure 12b,c.

### 8.5. Results with Definitive Tension

**Figure 13:**
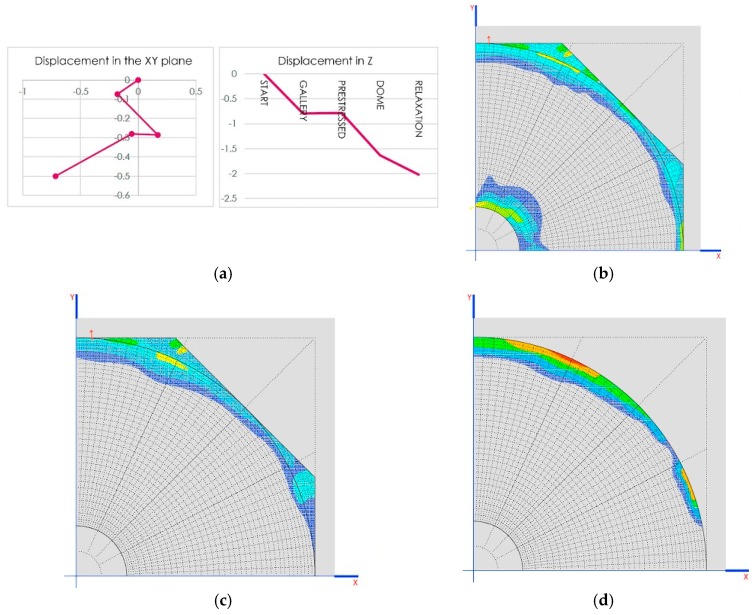
(**a**) Diagram of the displacement of the connecting rod support (mm); (**b**) traction in the dome (MPa) in the penultimate phase of construction; (**c**) tractions in the dome (MPa) in the last phase of construction; (**d**) behavior of the dome without the tympanums working.

It is shown in Table 20, Table 21 and Table 22 the different results of displacement, axial forces and compression in arches:

Traction on the dome (MPa) in the two final phases is shown in Figure 13b,c.

Observing the dome without the tympanums is shown in Figure 13d.

**Figure 14:**
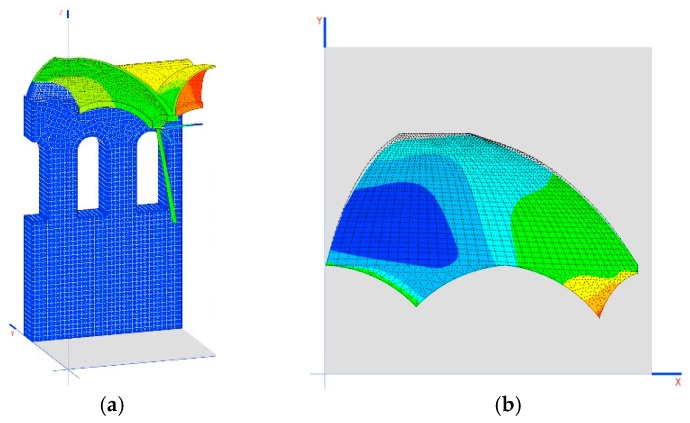

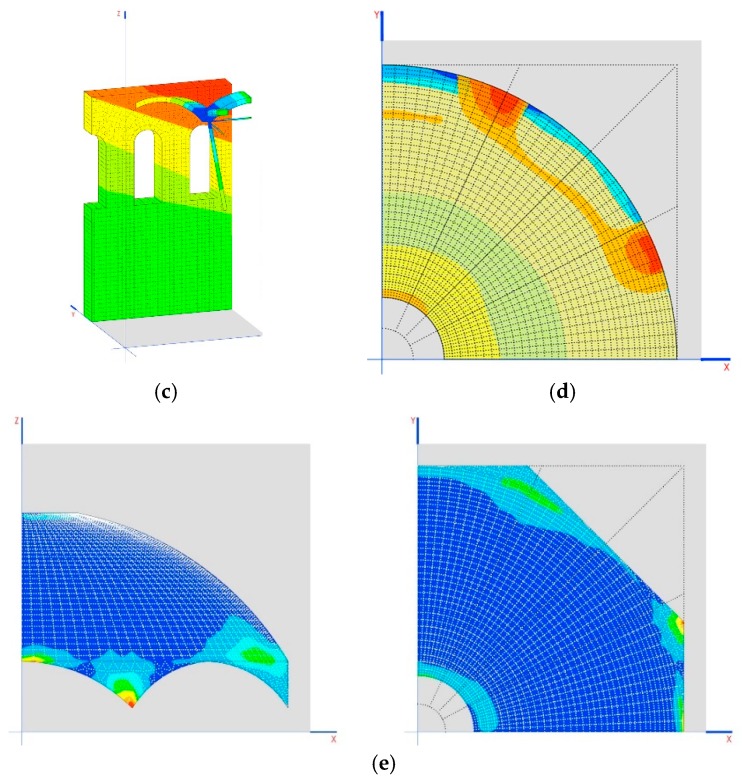
(**a**) Displacement in Z of the building; (**b**) detail of the dome and its deformation (the blue zone is the one supported on the wall, the green zone is the one supported on the gallery); (**c**) displacement in Y of the wall in mm (perpendicular to its plane); (**d**) main directions of stresses in the dome; (**e**) tension peaks in the intersection of the arches.

The construction process alleviates the tensions on the dome by up to 0.6 MPa (without considering small amounts of 1 MPa on the tympanums), especially in sensitive areas such as the skylight, where the traction practically disappears (Figure 14a–e).

## 9. Resulting Tensions and Forces

### 9.1. Tensions in the Dome

**Figure 15:**
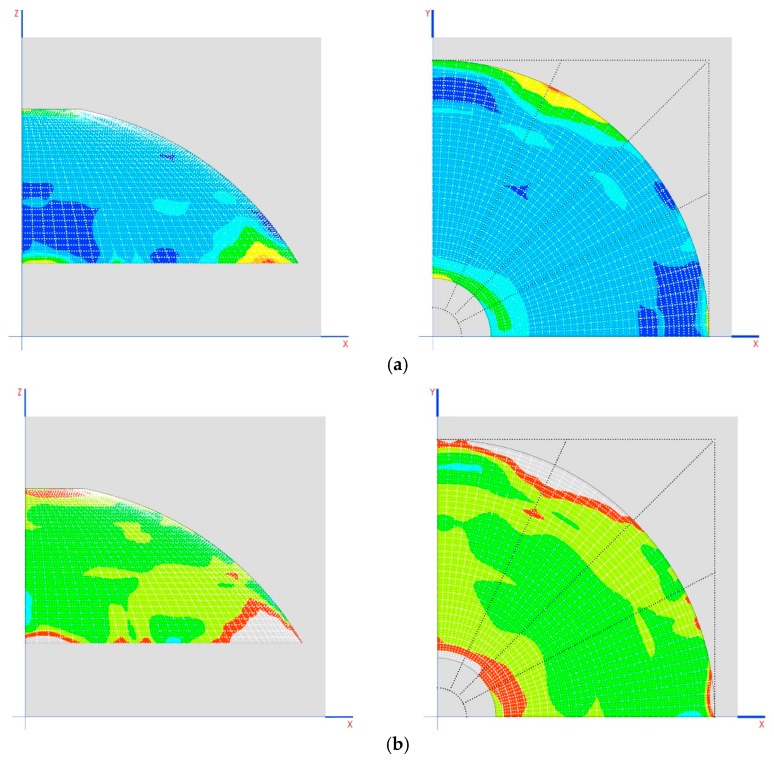
(**a**) Possible appearance of cracks without affecting the stability of the structure; (**b**) transparent areas are the most stressed.

Small amounts of tension of 2 MPa appear in the area where the arches meet, below the characteristic traction resistance of the material (5 MPa) (Figure 15a).

The appearance of cracks is expected in said areas, which allow tension in the dome to become relaxed at these points, without affecting stability of the structure. As can be seen in Figure 15, the structure remains in a state of tension that is far from breaking the stone.

**Figure 16:**
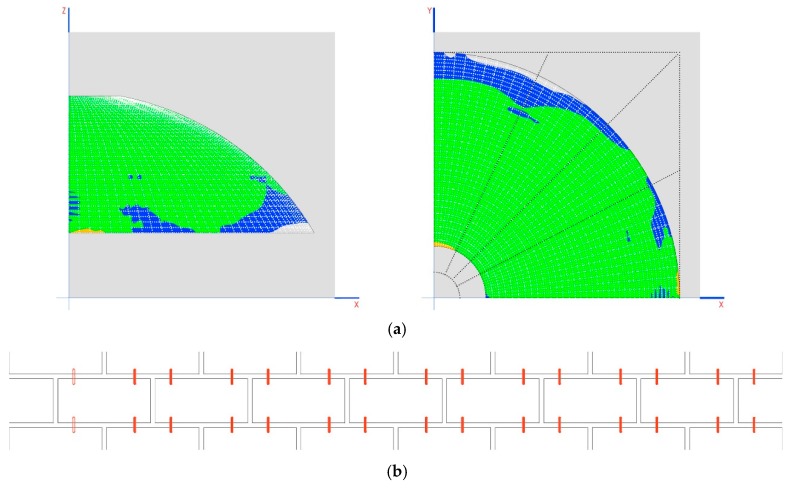
(**a**) Main tension is in the direction of the horizontal plane; (**b**) increased tensile strength with metal connectors placed as shown.

The breaking limit for mortar has been taken as 0.3 MPa. The areas that appear as having the greatest tension are shown in a transparent color in Figure 15b. The main tension is in the direction of the horizontal plane, with a maximum of 0.5 MPa (Figure 16a).

To increase compression in said area, increasing the thickness of the dome may be investigated. To increase the traction capacity of the structure, metallic connectors may be placed between the stone walls that connect the rows between inclines (Figure 16b).

### 9.2. Tensions on the Vault

**Figure 17:**
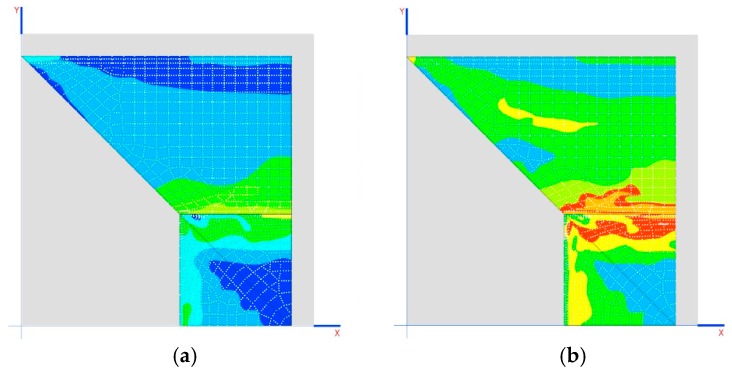
(**a**) The tension in the vaults are of the order of 2 MPa, considering the same conditions as for the dome; (**b**) the possibility of cracks in the direction of the gallery for tensioners greater than 0.3 MPa.

The tensions on the vault are the same as appear on the dome, namely, 2 MPa, and the same factors are therefore established as for the dome (Figure 17a).

Tensions greater than 0.3 MPa will also generate openings of cracks, this time in the direction of the gallery (Figure 17b).

In the case of the vault, we could try changing its geometry, reducing its bend, and proceeding with developments of 90° to 60° or lower, so that a larger lateral impact is produced, which would be beneficial for compensating the impacts of the dome, and would increase compression of the stone walls in the area that makes contact with the arches.

Due to the low tension produced, it is also estimated that the cracks generated would not affect the overall stability of the structure.

### 9.3. Tensions on the Walls

**Figure 18:**
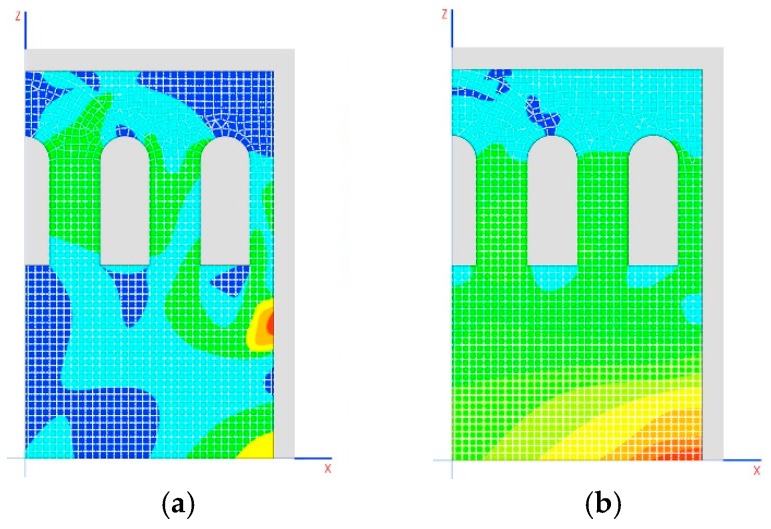
(**a**) Tension in the wall only to compression; (**b**) no noticeable traction stresses at any point on the wall.

The tension on the wall is completely under compression, with a maximum tension on the connecting rod support of 1.56 MPa (Figure 18a).

In no case does significant traction tensions appear (maximum 0.06 MPa below the mortar limit). (Figure 18b).

### 9.4. Stress on the Arches

**Figure 19:**
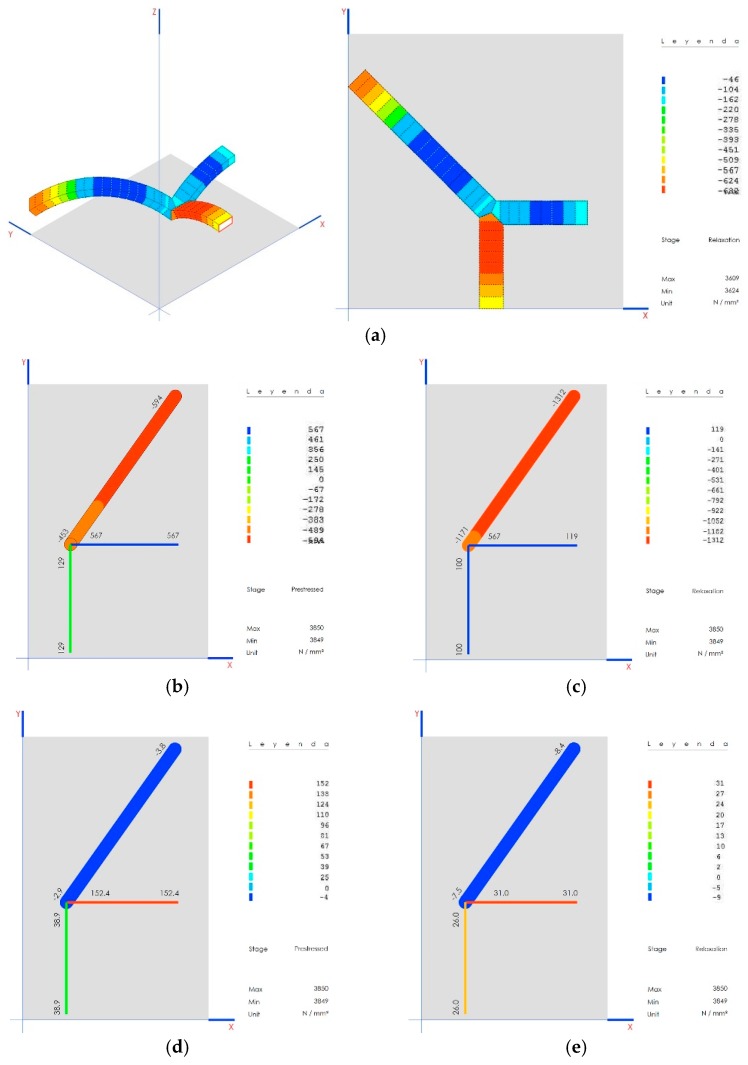
(**a**) The stability of the structure is guaranteed between 45 kN and the 624 kN because in that range the arches work in compression completely; (**b**) construction phase of prestressing; (**c**) final construction phase; (**d**) elastic limit in the prestressing phase of construction; (**e**) elastic limit in final construction phase.

Upon completion, the arches work under compression, within a range of 45 kN to 624 kN, which guarantees stability in the structure (Figure 19a).

### 9.5. Stress on the Connecting Rods and Crosspieces

The stresses on these elements vary significantly depending on the construction phase, as shown for prestressed (Figure 19b) and final (Figure 19c). These remain much lower than the elastic limit at all times (175 MPa): prestressed, Figure 19d; final, Figure 19e.

The connecting rod under compression reaches a maximum weight of 1312 kN when joined with the wall, staying far from its expected flexing load of 4800 kN.

The system works with the indications from axillary forces expected for each type of element at all times, which guarantees the stability of the structure.

The system of connecting rods and crosspieces has been considered as being jointed with concurrent forces. The connection element of the connecting rod must be studied in detail, which appears to be drawn as a swivel and is modeled as such, but it has a complexity that deserves special attention and study.

## 10. Conclusions—Viability of the Structure

The building is viable with the suggested system of connecting rods and crosspieces to bear the weight of the roof.

In accordance with the results obtained from a linear analysis for the construction process as indicated in the previous paragraph, all of the structural elements work as they should, namely:

- Arches under compression;
- Dome with meridians under compression;
- Crosspieces under traction;
- Connecting rod under compression.

The tension that appears on the dome during linear calculations slightly exceeds what is acceptable (1.0 compared to 0.3 MPa), but non-linear calculations show that these tensions relax with plastic distortions, due to the fact that the movements the structure must make are very small.

A system of connecting rods and crosspieces was analyzed. It was conceived to carry the vertical loads of a roof from a lower gallery towards its lateral walls.

This system raises several issues:

Shear failure on the wall—weight that is normally distributed will be concentrated towards a concrete point on the vertical surface.
Stability of the dome—due to the presumed system flexibility, excessive movements may make a structure as sensitive as a dome unable to work properly.
Final dimensions—the smelting elements must have achievable dimensions, at least from a technical point of view.

In light of the results obtained for tension on the wall and the dome, we have arrived at the conclusion that, in the case of compression, the elements made from rocky material are far from breaking (6 MPa) and for traction, they only reach a value of 0.8 MPa, which shows that the system can clear up to 0.3 MPa (mortar limit) through movements in plastic systems.

It should also be pointed out that the traction presented only occurs at very localized points of the structure, and is not general, so it could be said that the way this material works under compression is correct.

The metallic elements are divided into connecting rods and crosspieces. The connecting rods only present compression loads, staying far from the critical flexing weight, so they are stable.

As for the crosspieces, they have loading and unloading phases, with the loading process being understood as traction. Appropriate prestressing will prevent these elements from being subjected to compression, as they become bent due to their slimness and would put the integrity of the building in danger.

In general, the structure would benefit from an appropriate tension process, but a correct construction process is also key, which could occur in three phases:

1. Construction of load-bearing walls, supporting arches, crosspieces and connecting rods, and main arches.
2. Crosspiece tensioning. The crosspieces make the arches carry a compression load. This process can be carried out thanks to the weight supported by the main arches; without these, the tension would be very limited.
3. Construction of the dome. Due to the movements induced by it, the arcs will undergo an elongation process, substantially discharging their compression, which will be compensated with the previous testing process.

A fourth phase has been defined, which consists of partially unloading the longitudinal arch in the gallery, as it was observed that this action caused compressions in the other arches, clearly favoring the general structure.

The structure of connecting rods and crosspieces is a viable alternative to the classic gallery pillars, which carry the vertical loads directly to the foundations.

The presented system works together with an arch, which carries the loads to its brackets—in this case, the lateral walls of the building.

Due to the point where the connecting rods are expected to be anchored (lower than originally planned), the lower transversal crosspiece will be dispensable, as long as the anchorage point on the wall has sufficient resistance for loads that are perpendicular to its plane.

## Figures and Tables

**Table 1:** Evaluation of construction materials for execution of the calculation models.

	Natural Rock	Mortar	Ductile Cast Iron
Young’s modulus, E (GPa)	50	-	175
Elastic limit, fyk (GPa)	-	-	175
Resistance under compression, fck (MPa)	50	7.5	-
Resistance under traction, ftk (MPa)	5	0.3	-
Density, d (kN/m^3^)	28.5	-	75
Thermic coefficient, a (°C^−1^)	-	-	1.2 × 10^5^
Cohesion, c (MPa)	-	0.75	-
Angle of internal friction, ϕ (°)	-	68	-

**Table 2:** Resulting force on the support.

Fx	Fy	Fz
−170 kN	−186 kN	1205 kN

**Table 3:** Components of the reaction in different directions.

Angle	Value	FX’	FY’	FZ’
Arches starting	45°	−31.3	−674.9	1029.1
Development of dome	60°	−31.3	−385.6	1168.7
Resultant	78°	−31.3	0	1230.6

**Table 4:** Dimensions of the connecting rod.

Bx	By	Bz	Length
3.935 m	5.569 m	11.81 m	13.68 m

**Table 5:** Z component and length when the connecting rod forms an angle of 59.9949° with the horizontal plane.

Bz	Length
11.808 m	13.635 m

**Table 6:** Wall displacement in the support area of the dome.

Dx	Dy	Dz
0.000 mm	0.215 mm	−0.147 mm

**Table 7:** Reactions in support after imposed displacements.

Matrix K	FX (kN)	FY (kN)	FZ (kN)
DX = 1 mm	2068	−526	−727
DY = 1 mm	−526	1992	67
DZ = 1 mm	−727	67	1189

**Table 8:** Displacement of crosspiece if shortened by 1 mm (537 kN).

	FX (kN)	FY (kN)	FZ (kN)	DX (mm)	DY (mm)	DZ (mm)
Crosspiece 1	537	0	0	0.35411	0.08639	0.21533
Crosspiece 2	0	−537	0	0.08839	0.28916	0.03656

**Table 9:** Forces on arches with crosspieces of 120 mm.

Load Case	ARCH 1 (kN)	ARCH 2 (kN)	ARCH 3 (kN)
Self weight	2.0	80.1	5.1
Prestressed_1	145.3	−81.5	18.8
Prestressed_2	76.0	17.7	−64.4

**Table 10:** Forces on arches with crosspieces of 40 mm.

Load Case	ARCH 1 (kN)	ARCH 2 (kN)	ARCH 3 (kN)
Self weight	−64.7	116.4	−2.0
Prestressed_1	21.1	−12.8	3.9
Prestressed_2	8.5	3.8	−9.4

**Table 11:** Displacement in the support between connecting rod and arches.

	DX (mm)	DY (mm)	DZ (mm)
Crosspiece of 120 mm	−0.35	0.05	−1.31
Crosspiece of 40 mm	−0.5	0.02	−1.39

**Table 12:** Comparison of the tensions.

	Tension in the Dome	Force on the Arches
Maximum traction 45°	1.596 MPa	505 kN
Maximum traction 60°	0.851 MPa	186 kN

**Table 13:** Displacement in the support between the connecting rod and arches.

	DX (mm)	DY (mm)	DZ (mm)	Total (mm)
At 45°	−0.85	0.13	−2.42	2.57
At 60°	−0.35	0.05	−1.31	1.36

**Table 14:** Forces in different load cases.

Load Case	ARCH 1 (kN)	ARCH 2 (kN)	ARCH 3 (kN)
Self weight	−64.7	116.4	−2.0
Prestressed_1	21.1	−12.8	3.9
Prestressed_2	8.5	3.8	−9.4

**Table 15:** The influence matrix loads on arches 2 and 3.

	40 mm Crosspieces		120 mm Crosspieces	
	ARCH 2 (kN)	ARCH 3 (kN)	ARCH 2 (kN)	ARCH 3 (kN)
Self weight	305.5	−184.5	227.6	−145.4
TEN_X (1 mm)	−60.8	0.3	−435.9	9.6
TEN_Y (1 mm)	1.7	−84.5	16.2	−535.2

**Table 16:** The influence matrix loads on arches 2 and 3 with 70 mm crosspiece, a rigidity of 171 kN/mm and a weight limit of 675 kN.

	70 mm Crosspiece	
	ARCH 2 (kN)	ARCH 3 (kN)
Self weight	283.1	−172.1
TEN_X (1 mm)	−174.1	1.7
TEN_Y (1 mm)	5.3	−230.2

**Table 17:** Displacement in mm of the anchorage point of the connecting rod with the arches (Figure 12a).

Phase	DX	DY	DZ
Gallery	−0.179	−0.073	−0.795
Prestressed	0.170	−0.286	−0.782
Dome	−0.056	−0.280	−1.634
Relaxation	−0.644	−0.328	−1.977

**Table 18:** Axial forces in kN on connecting rods and crosspieces.

Phase	Connecting Rod	Crosspieces 1	Crosspieces 2
Gallery	−438.2	30.7	−12.5
Prestressed	−438.5	585.1	153.1
Dome	−1019.3	623.8	154.0
Relaxation	−1106.0	110.3	−56.1

**Table 19:** Axial forces in kN on arches.

Phase	ARCH 1	ARCH 2	ARCH 3
Gallery	0	−172.13	283.06
Prestressed	0	−437.83	−335.54
Dome	93.64	−435.08	−298.04
Relaxation	−104.55	−439.68	−200.37

**Table 20:** Displacement of the connecting rod support (mm) (Figure 13a).

Phase	DX	DY	DZ
Gallery	−0.179	−0.073	−0.795
Prestressed	0.170	−0.286	−0.782
Dome	−0.056	−0.280	−1.634
Relaxation	−0.717	−0.501	−2.025

**Table 21:** Axial forces in connecting rod and crosspieces (kN).

Phase	Connecting Rod	Crosspieces 1	Crosspieces 2
Gallery	−438.2	30.7	−12.5
Prestressed	−438.5	585.1	153.1
Dome	−1019.3	623.8	154.0
Relaxation	−1063.2	122.6	116.4

**Table 22:** Compression in arches (kN).

Phase	ARCH 1	ARCH 2	ARCH 3
Gallery	0	−172.13	283.06
Prestressed	0	−437.83	−335.54
Dome	93.64	−435.08	−298.04
Relaxation	−74.42	−461.84	−189.3
